# New data on Weddell seal (*Leptonychotes weddellii*) colonies: A genetic analysis of a top predator from the Ross Sea, Antarctica

**DOI:** 10.1371/journal.pone.0182922

**Published:** 2017-08-10

**Authors:** Ighor Antunes Zappes, Anna Fabiani, Valerio Sbordoni, Arnold Rakaj, Roberto Palozzi, Giuliana Allegrucci

**Affiliations:** Department of Biology, University of Rome Tor Vergata, Rome, Lazio, Italy; National Cheng Kung University, TAIWAN

## Abstract

In this paper, we studied the genetic variability in Weddell seal from colonies in Terra Nova Bay and Wood Bay, both sites located in the Ross Sea area, Antarctica. Two mitochondrial genes and one nuclear gene, with different mutation rates, were sequenced to investigate the haplotype diversity of the colonies and to test for a possible recent expansion. Fifteen microsatellites were used to analyze their genetic structure. Sequenced genes and microsatellites were also used to estimate the effective population size of the studied colonies and the Ross Sea seal population. The Ross Sea has a high density population of Weddel seals, with an estimated effective number of 50,000 females, and 1,341 individuals for the sampling area, possibly due to its high primary production. The colonies showed high diversity (*Hd* > 0.90) and many exclusive haplotypes (> 75%), likely a consequence of the surprisingly high site fidelity of Weddell seals, despite the proximity of the colonies. Nevertheless, there was low microsatellite differentiation between colonies, suggesting that they are part of a single larger population. Their expansion seemed to have started during the last glacial cycle (around 58,000 years ago), indicating that the Ross Sea seal populations have been present in the area for long time, probably due to the lack of hunting by humans and terrestrial predation. As a top predator, the role of Weddell seals in the Ross Sea ecology is crucial, and its demographic dynamics should be monitored to follow the future changes of such an important ecosystem.

## Introduction

Weddell seal (*Leptonychotes weddellii*) is part of the tribe Lobodontini, also collectively called Antarctic seals [1], and inhabit the coastal areas of Antarctica. Among them, the Weddell seal shows the most southern distribution and breeds on fast-ice. It is a long-lived mammal, with an average life span of 25 years and females generally living two to three years longer than males. Adult seals are large, reaching 2.5–3.5 m of length and weighing 400–600 kg, with females slightly bigger than males [2]. They occupy a high trophic level in the food web of Antarctica [3], and they have no terrestrial predators. As they inhabit places with only little or no anthropogenic disturbance [4], their historic population changes should be a reflection of only natural processes. As a result, their colonies can be large, and the entire Antarctic population has estimated to be around 730,000 to 800,000 individuals [3], [5]. However, a simple counting of the total number of seals may not reflect the number of individuals that are actually contributing to the next generations and thus the long-term evolutionary potential of the population. A more accurate parameter would be the genetic effective population size (*N*_*e*_), which represents the number of individuals in an ideal population that would have the same rate of genetic drift as the observed population [6]. In breeding colonies of *L*. *weddellii* there is some level of variation in male reproductive success, mating is not random, adult sex ratio can differ from 1:1, and population size may change across generations. All these factors can drastically reduce *N*_*e*_ [7], as it has already been shown, through the analysis of mitochondrial genes, in a colony from McMurdo Sound in the Ross Sea, where a *N*_*e*_/*N* ratio of around 0.146 was found [4].

The mating system of Weddell seals is classified as aquatic, because copulation always occurs under water, even if they breed on land and close to the shore. Their mating system is poorly understood, due to the inherent difficulties in observing reproductive behavior that occurs below the surface [8]. During the austral spring season, from October to December, females haul out onto the fast ice to give birth close to tidal cracks, usually one pup per season [9–12]. The nursing period lasts for about 40 days [13]; however, after two or three weeks of initial nursing, the mothers begin to forage for short periods of time, going under water through small holes in the ice, and then returning to continue lactating [11], [14]. Females breed soon after pup weaning (around six weeks from parturition), while they are still close to their breeding colony [13]. As seasonal breeders, females gather together both spatially and temporally, and males are able to increase their mating success and breed with more females [15]. Their potential for polygyny is high, as there is no need for paternal care, and the environmental structure of the fast ice forces females to aggregate [16], [17]. However, their actual level of polygyny is moderate when compared to other seals [18], [19] and the harems are usually small, up to five females per male [20]. Environmental instability also limits the study of population dynamics in this species, as the observation of a large number of individuals over a long period and on a regular basis is almost impossible. In this context, molecular markers, such as microsatellite genotyping and mitochondrial DNA sequences, can be very useful, as they allow the estimation of genetic diversity, relatedness of individuals, population genetic structure and affiliation [21].

Weddell seals can show a significant level of site fidelity, as both males and females tend to return to the same breeding site for several consecutive reproductive seasons [2], [22]. Nevertheless, as the fast ice is not as stable as the mainland, its dynamics decrease the level of site fidelity when compared to other pinnipeds [17], [23–25]. In fact, colonies of Weddell seals inhabiting areas close to each other show significant gene flow, while those with no physical connections revealed higher population structure and only little evidence of long-distance migration [1], [26].

**Fig 1:**
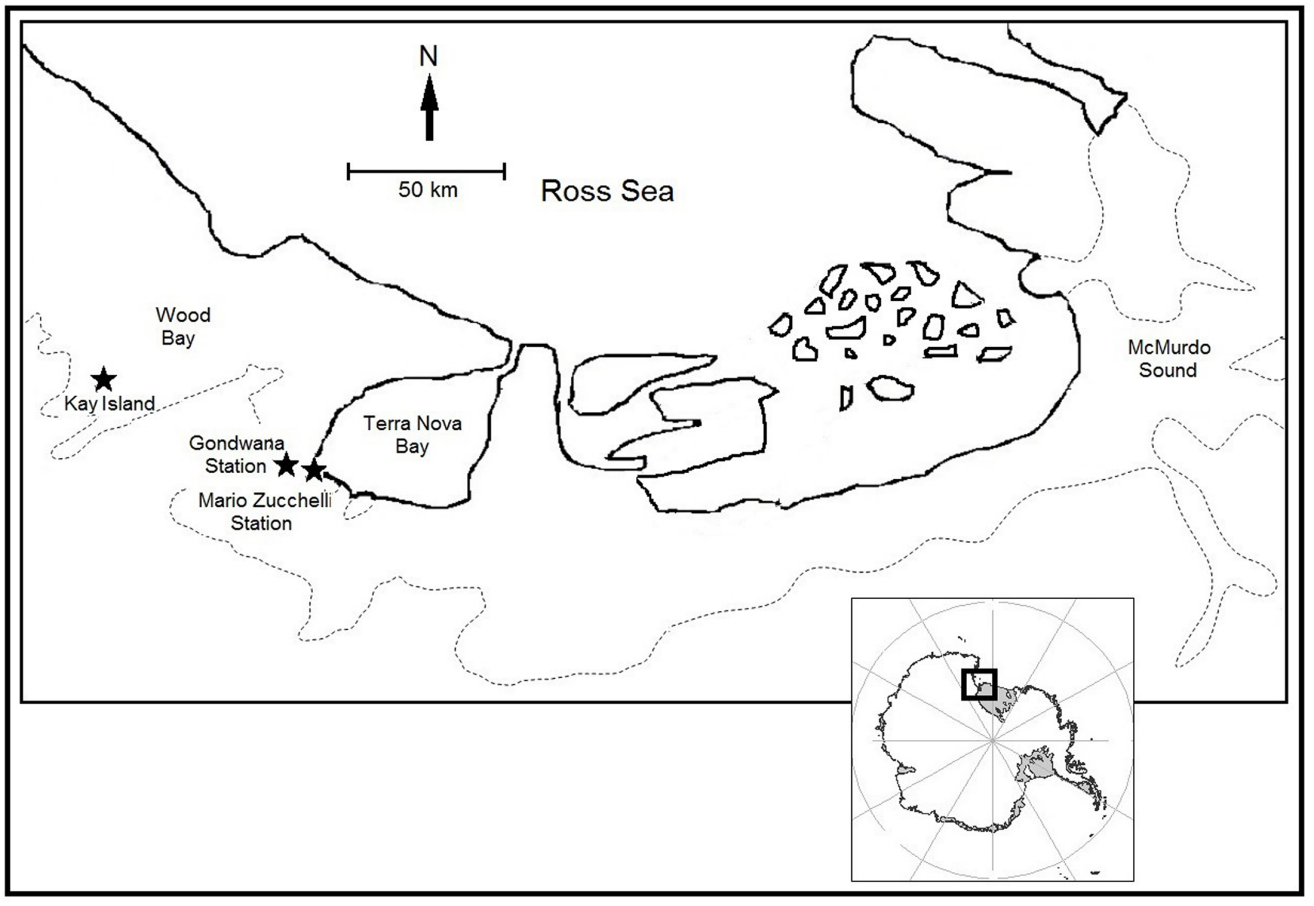
Map of the sampling areas. Dash lines represent the grounding line.

Since the late 60’s, *L*. *weddellii* has been mainly studied in McMurdo Sound, an area that connects the Ross Sea with the Ross Ice Shelf cavity (Fig 1) [27], and the study represents one of the best examples of long-term studies on mammals. Nevertheless, the site around the American base has a peculiar ecological setting, and the lack of replication in other colonies might not allow the application of the results on a more general scale. In particular, along the coast of Victoria Land, west of McMurdo Sound, is Terra Nova Bay (Fig 1), which has a diverse range of ecological features and has never been studied before. As this area is often ice-free and has been characterized by a persistent polynya (i.e., permanent open water surrounded by sea ice) since the beginning of the Holocene [28], it is more accessible to seals and richer in nutrients. Areas with characteristics like Terra Nova Bay, together with philopatry (i.e., return to natal site to breed) and site fidelity for this species, can represent a perfect scenario for the evolution of genetic structure among breeding colonies.

In this context, our main goal was to improve our knowledge of the genetic dynamics and genetic history of Weddell seal colonies, and to infer Antarctic landscape changes in the recent past. The colonies studied here inhabit two localities of the Ross Sea never before studied: Terra Nova Bay and Wood Bay. Both areas belong to the northern part of Victoria Land, around 300 km from McMurdo Sound. In particular, we analysed the genetic structure between colonies, their population expansion dynamics during the Quaternary, and their effective population size. The results were analysed and discussed in the context of recent climate changes and landscape history of the entire Ross Sea.

## Materials and methods

### Sampling

Our study area identified two breeding colonies of Weddell seals in the Ross Sea: one occupying the inner part of the ice-free Terra Nova Bay (TB), and the other located 90 km north, in Wood Bay (WB). In the first expedition (November/December 2012), 29 scat samples were collected in TB, within a small area around the Italian Research Station “Mario Zucchelli” (coordinates 74°41′38''S, 164°06′53''E). Faecal material was conserved at -20°C until DNA extraction. In the second expedition (November/December 2014), skin samples were collected from the hind flippers of seals breeding in the same area and around the German Research Station “Gondwana” (coordinates: 74°38′7''S, 164°13′17''E, TB). In addition, 74 skin samples were collected in WB, on Kay Island (coordinates: 74°04.048'S, 165°19.293'E). Skin samples were collected by direct seal handling and preserved in 100% ethanol or frozen at -80°C, until DNA extraction. The sampling protocol used in this study caused only a little (and irrelevant) disturbance to the seals, and was approved by the Italian Ministry of Foreign Affairs, referring to the PNRA Research Project 2013/AZ.01, according to the "Protocol on Environmental Protection to the Antarctic Treaty", Annex II, art.3. Localities are shown in Fig 1 and samples are listed in Table 1.

**Table 1:** Samples and localities.

Locality	Year	Coordinates	Males	Females	Pups	Total	Sample type	Colony
Mario Zucchelli Station	2012	74°41′38''S 164°06′53''E	?	?	?	29	Scat	Terra Nova Bay
	2014	74°41′38''S 164°06′53''E	1	2	0	3	Skin and muscle	
Gondwana Station	2014	74°38′7''S 164°13′17''E	7	11	6	24		
Kay Island	2014	74°04.048'S 165°19.293'E	6	27	41	74		Wood Bay

### DNA extraction

Total DNA (tDNA) was extracted from all samples. For the scat samples, we used a protocol with silica spin columns from the QIAamp^™^ DNA stool mini kit (QIAGEN). For the skin samples, the extraction was done with a standard phenol-chloroform technique with some modifications [29]. In order to assess the DNA quality, 3 μL of each extracted sample was visualized in 1% agarose gels with ethidium bromide.

### Mitochondrial and Y chromosome DNA

We amplified two sequences from mitochondrial DNA and one from the Y chromosome. The first mtDNA sequence was a 446 bp fragment of the gene responsible for the synthesis of the protein Cytochrome b (*CYB*). The primers used were *mcb398* forward (5'-TACCATGAGGACAAATATCATTCTG-3') and *mcb869* reverse (5'-CCTCCTAGTTTGTTAGGGATTGATCG-3') [30]. The 20 μL reaction volume contained: 1.6 μL tDNA, 8 pmol of each primer, 2 mM of MgCl_2_, 0.6 U of *Taq* polymerase (Thermo Scientific^™^), 0.2 mM of each dNTP, reaction buffer (Thermo Scientific^™^). The conditions for the amplification were: initial denaturation at 95°C for 5', 35 cycles of 95°C for 30'', 58°C for 45'' and 72°C for 30'', and a final extension at 72°C for 10'.

The second mtDNA sequence was a 456 bp fragment of the control region of D-loop (*DLOOP*). The primers used were forward *TDKD* [31] (5′-CCTGAAGTAGGAACCAGATG-3') and reverse *L15926* [32] (5′-TCAAAGCTTACACCAGTCTTGTAAACC-3'). The 12.5 μL reaction volume contained: 1 μL tDNA, 4 pmol of each primer, 2 mM of MgCl_2_, 0.6 U of *Taq* polymerase (Thermo Scientific^™^), 0.2 mM of each dNTP, reaction buffer (Thermo Scientific^™^). The conditions for the amplification were: initial denaturation at 95°C for 2', 40 cycles of 95°C for 30'', 48°C for 45'' and 62°C for 1.5', and a final extension at 72°C for 5'.

On the Y chromosome, we sequenced a 874 bp fragment of the sex-determining region on Y (*SRY*). The primers used were forward *USRY1F* [32] (5′-GTATCCAGTGGTGTTTTAATAGC-3') and reverse *USRY1R* [32] (5′-GCAGCCATAAACCCAGACTG-3'). We also performed semi-nested reactions with the internal primers *USRY2F* (5′-GTGGTGTTTTAATAGCTAGTAG-3') and *USRY2R* (5′-CCAGACTGAATCAATTCAG-3'). All primers were obtained from Nakagome et al. [33]. The 25 μL reaction volume contained: 1 μL tDNA, 4 pmol of each primer, 2 mM of MgCl_2_, 0.6 U of *Taq* polymerase (Thermo Scientific^™^), 0.2 mM of each dNTP, reaction buffer (Thermo Scientific^™^). The conditions for the amplification were: initial denaturation at 94°C for 5', 40 cycles of 94°C for 1', 55°C for 1'' and 72°C for 1.5', and a final extension at 72°C for 10'.

For the purification of mitochondrial PCR products, we used the enzymatic clean-up ExoSAP-IT^™^ (Affymetrix), following the standard protocol. All PCR products were packed to the Center for Genetic Analyses of Biodiversity in Yale University, USA, where they were sequenced in both directions on a Big Dye Terminator^™^ sequencer. The Y chromosome, *CYB* and *DLOOP* sequences were aligned with software MEGA (v. 6.0) [34], after checking the chromatograms for reading errors and manually editing them, if needed. Number of haplotypes (*h*), nucleotide diversity (*π*) and haplotype diversity (*Hd*) were assessed using the software DnaSP (v. 5) [35].

We implemented network analyses with the three sequences, in order to reconstruct intraspecific relationships and specificity of haplotypes, using the neutral coalescent theory statistic [36]. For mtDNA networks, we excluded pups with a known mother, in order to avoid a bias in the haplotype frequencies. For the Y chromosome DNA network, no male pups had a known father, so we used all individuals. We used a median joining algorithm with probabilities above the 0.95 limit [37], as implemented in the software Network^©^ (v. 5.0.0.0, Fluxus Technology Ltd). We also built an extra network for the *DLOOP* fragment, including 84 haplotypes from the Weddell seal colony of McMurdo Sound (MS), obtained from Curtis *et al*. [4]. Since we did not know the frequencies of the MS haplotypes, this extra network was only used to check if there were exclusive and/or shared haplotypes among the three colonies.

The seal colonies were checked for possible expansion with two different approaches. First, with the program Arlequin (v. 3.0) [38], we analysed the distribution of pairwise haplotype differences, (i.e., mismatch distribution), which tends to be unimodal and smooth if the population size has changed only in the last generations. Within the same program, the sum of squares can also confirm sudden expansion or stationary population size. Then, we estimated the number of generations since the beginning of the expansion (*t*) using the formula *t* = *Τ* / 2*u* [39], where *Τ* is the peak of the distribution and *u* is the cumulative (across the sequence) probability of substitution. This software was also used to perform raggedness statistics [40], which measures the smoothness of the mismatch distribution, and presents lower values for population under growth model. In addition, with the program DnaSP we performed a Fu's test of selective neutrality, which is based on the differences between expected and observed numbers of alleles [41] and is very sensitive to population demographic expansion (which usually leads to large negative *F*_*s*_ values).

We used Migrate (v. 3.6) [42] to obtain maximum likelihood estimates *F*_*st*_ (*Θ*) for both colonies. Migrate uses a random seed to initiate calculations, so replicate estimates of *Θ* vary. Ten trials were performed for each colony and the median values were used. Parameter Θ was also obtained from LAMARC (v. 2.0) [43], with a Bayesian, MCMC maximum likelihood approach [44]. This analysis was run using an initial burn-in period of 10,000 iterations, followed by runs of 200,000 steps and taking into account nucleotide differences among haplotypes. The parameter obtained was used to calculate *N*_*e*_, but with the adapted formula *N*_*e(f*,*m)*_ = *Θ* / 2*μ*, where *N*_*e(f*,*m)*_ is the effective population size of females (for mtDNA) or males (for Y chromosome DNA), and μ is the mutation rate for each fragment. This formula variation is used for genes with one parent heritage, such as mitochondrial or Y chromosome fragments. Estimation for *N*_*e(f*,*m)*_ were calculated using mutation rates (*μ*) obtained from literature. For *SRY*, a general rate for mammals was established as 2.8 x 10^−8^ substitution / site / year by Nagai [45]. However, as Y chromosome genes evolve fast, we preferred to use the specific rate of Carnivora found by King *et al*. [46]. Our *CYB* rate was 3.0 x 10^−8^ substitution / site / year, from two studies: Pesole *et al*. [47] did evolutionary analyses for each functional DNA region for two seal species (*Phoca vitulina* and *Halichoerus grypus*), and considered synonymous and non-synonymous codon positions separately, in the protein-coding genes; in Nabholz *et al*. [48], the authors used an extensive *CYB* data set (also from fossil data), measured the lineage-specific mitochondrial mutation rate across 1,696 mammalian species (131 carnivores) and compared it with the mutational nuclear rate.

For the *DLOOP* analysis, we used the mutation rate of 7.5 x 10^−8^ substitution / site / year, from Slade *et al*. [49]. They estimated the value using *DLOOP* divergence and fossil records of northern and southern elephant seals, leopard seal and Weddell seal. Using their estimate, our data did not show tranversions saturation of nucleotides up to 30 million years of *DLOOP* divergence among populations.

As a value for the generation time was also needed, we used an estimate of nine years, according to Croxall *et al*. [50] and Hadley *et al*. [51].

### Microsatellites

We used 17 polymorphic microsatellite markers characterized from Antarctic seals [52] to screen all our seals. Names of microsatellites and primers used are listed in Table 2. For each marker amplification, the 12.5 μL reactions contained: 1 μL tDNA, 4 pmol of each primer, 2 mM of MgCl_2_, 0.6 U of *Taq* polymerase (Thermo Scientific^™^), 0.2 mM of each dNTP, reaction buffer (Thermo Scientific^™^). All loci were amplified according to the following conditions: initial denaturation at 95°C for 5', 35 cycles of 95°C for 30'', 51–58°C for 45'' and 72°C for 30'', and a final extension at 72°C for 7'. The forward primer of each marker was fluorescently labelled with one of 6-FAM, NED^™^ or HEX (ABI) for future readings.

**Table 2:** Features of 17 microsatellite loci for Antarctic seals.

Locus	Size range	Motif	Primers
HL2	110–114	(GT)9C(GT)5	F:**H**CAAACACCACTATTTCCCT
			R:AGGTTGTGGTCTGAAGAAT
HL4	125–133	(GT)12	F:**N**GCTAAAAGCATCTCCTTACC
			R:CGGCATAGAAATCTTTTACA
HL8	99–121	(GT)17(T)2(G)7	F:**H**CACAGGGATTAGGGGAAAG
			R:AGCCTTAAAAGTTGTCTAT
HL14	230–236	(GT)18	F:**H**GACCTGAGCTGAAGGCAGAC
			R:GTTTGTTCAGTGTGTCCATTGTAGTTAC
HL15	119–139	(GT)14	F:**H**CATCTTGTAGTGCCAAAAAC
			R:ATCTTTCAGTTGACCCTTCT
HL16	134–140	(GT)13	F:**H**CACTTATCTCGCCCTATATCCA
			R:CAGCCACAGCCAACACAA
HL20	93–125	(GT)20	F:**H**CTCAACACAGGCGTAATATTG
			R:GATCTTTGACAAGGAGAGTATGTT
LC5	153–167	(GT)9	F:**H**ATCTTCAGGCTTTCTTCT
			R:TTCACGGACTCAAATAAT
LC28	128–136	(GT)11	F:**H**TCATATAATACCCACCTCTGTAAG
			R:TGCCTCGTGATGAAAAACT
LW4	125–195	(GT)17	F:**N**TCCCAGAAGACCTACTCC
			R:ATTCCTTTCCTGCGTATC
LW7	159–173	(GT)16	F:**H**TGGGCTTTCTACAGTTC
			R:ACATAACTCAAGGGACAA
LW8	103–107	(GT)11	F:**F**CCTCTTTTCCTCTCTCTT
			R:CAATGTGGATGGAGTAAA
LW10	110–138	(GT)25	F:**F**AACACTAGCCCTGACTTC
			R:TTACAGAGCAGGAGTTCA
LW11	157–187	(GT)26	F:**H**CTCTCCCTCTCACCTTCC
			R:GGCAAATGAGGTGATGTC
LW15	138–152	(GT)11	F:**F**GATCTCTCTCTCTTTCAC
			R:CTGTAACTTCTCCAAACA
LW16	161–175	(GT)11	F:**F**CACTCCCCCACTGCTTGT
			R:ATTAGTTGCAATTTTGAGACACTC
LW20	122–146	(GT)20	F:**F**GACTCTTGCCCCCTTCAG
			R:GTTTCACAGACCTGCCTCTAGTG

Labelled primers are indicated with letters N, H or F (NED^™^, HEX or 6-FAM fluorescence, respectively).

All products were visualized with 1% agarose gel electrophoresis with ethidium bromide to assess the quality of the amplifications. We then added formamide for conservation. All PCR products were packed to the Center for Genetic Analyses of Biodiversity in Yale University, USA, where they were scanned on a 3730xl 96-Capillary Genetic^™^ analyser.

We investigated the maternity assigned on the field with the software Cervus (v. 3.0.7) [53]. The program compares the genotype of each individual pup to all adult females (specified in different files) and calculates the average number of loci with at least one shared allele. With the same program, we also compared unassigned pups with adult females, in order to find missed maternities, and considered a match mother-pup reliable when sharing one allele for at least 16 of the 17 markers, following the procedure of Gelatt [54]. Pups with detected mothers were then discarded from the following microsatellite analyses, because of the bias they could produce.

Genetic diversity was calculated as the number of alleles for each microsatellite locus in the two colonies. We estimated null allele frequencies with the software Genepop (v. 4) [55] and Hardy-Weinberg Equilibrium with Arlequin to check the quality of the microsatellites, after Bonferroni correction. In order to compare the proportion of the variance contained in the individuals, we calculated the inbreeding coefficient (*F*_*is*_) for each locus and across all loci for both colonies with the software Genetix (v. 4.05) [56], as well as the expected and observed heterozygosities (*H*_*e*_ and *H*_*o*_, repectively). Finally, the level of population genetic structure was estimated with *F*_*st*_ within and between colonies, and the geographic partition of microsatellite genetic variation was checked using a MCMC method with Genepop.

Population genetic substructure was investigated with the software Structure (v. 2.3) [57]. We used an admixture model with correlated allele frequencies and sampling information, as suggested by Hubisz *et al*. [58]. The program was run for *K* values from one to four (five runs for each *K*) using 500,000 MCMC repetitions and a burn-in of 100,000 steps. The most likely number of groups would correspond to the *K* value that shows the maximum increase in Ln [Pr (*X|K*)] over successive increases in *K*.

We estimated *N*_*e*_ for each colony, with one-sample methods used in the software LDN_e_ (v. 1.31) [59] and N_e_Estimator (v. 2.01) [60]. LDN_e_ estimates contemporary effective population size (*N*_*e*_) based on linkage disequilibrium data, and it calculates separate estimates excluding rare alleles. N_e_Estimator, instead, uses an improved implementation of bias correction for dealing with missing data. As a consequence, N_e_Estimator estimations will be generally lower than those from the other implementations.

## Results

### Genetic structure

For *CYB*, we obtained the target fragment (446 bp) from 71 and 26 seals from the colonies of WB and TB, respectively (GenBank Accession numbers KY594314-KY594410). The number of different haplotypes from each colony was 22 (24 polymorphic sites) and 16 (17 polymorphic sites), respectively. When analysed together, both colonies showed h = 31 (29 polymorphic sites). For *DLOOP*, we successfully sequenced the target fragment (456 bp) from 72 individuals from WB and 52 from TB (GenBank Accession numbers KY594262—KY594313, KY582597-KY582668), which showed *h* = 37 (59 polymorphic sites) and *h* = 26 (29 polymorphic sites), respectively. For both colonies together, *h* = 57 (67 polymorphic sites). For the Y chromosome gene *SRY*, the target fragment (874 bp) was amplified for 25 males from WB and eight males from TB (GenBank Accession numbers KY608912-KY608944). As the number of males from TB was too low, we analysed the colonies together. Nucleotide diversity of *SRY* gene was congruent with the mammal range (0.0001–0.001) [61–64]. Results on fragments variation are shown in Table 3.

**Table 3:** Genetic variation of WB and TB colonies.

Fragment	Index	WB	TB	WB + TB
*CYB*	*N*	71	26	97
	*h*	24	14	31
	*Tr/Tv*	35.39	18.06	8.53
	*Hd*	0.918 ± 0.016	0.940 ± 0.027	0.925 ± 0.014
	*π*	0.007 ± 0.003	0.007 ± 0.003	0.007 ± 0.003
	*PrH*	17	7	//
*DLOOP*	*N*	72	52	124
	*h*	42	27	57
	*Tr/Tv*	4.76	45.25	5.85
	*Hd*	0.976 ± 0.015	0.948 ± 0.020	0.960 ± 0.010
	*π*	0.013 ± 0.000	0.009 ± 0.004	0.011 ± 0.006
	*PrH*	30	15	//
*SRY*	*N*	//	//	33
	*h*	//	//	7
	*Tr/Tv*	//	//	0.89
	*Hd*	//	//	0.653 ± 0.003
	*π*	//	//	0.001 ± 0.000

Number of samples (*N*), number of haplotypes (*h*), Haplotype diversity (*Hd*), transition on transversion ratio (*Tr* / *Tv*), nucleotide diversity (*π*), Private Haplotypes (*PrH*). Average values are shown ± standard deviation.

**Fig 2:**
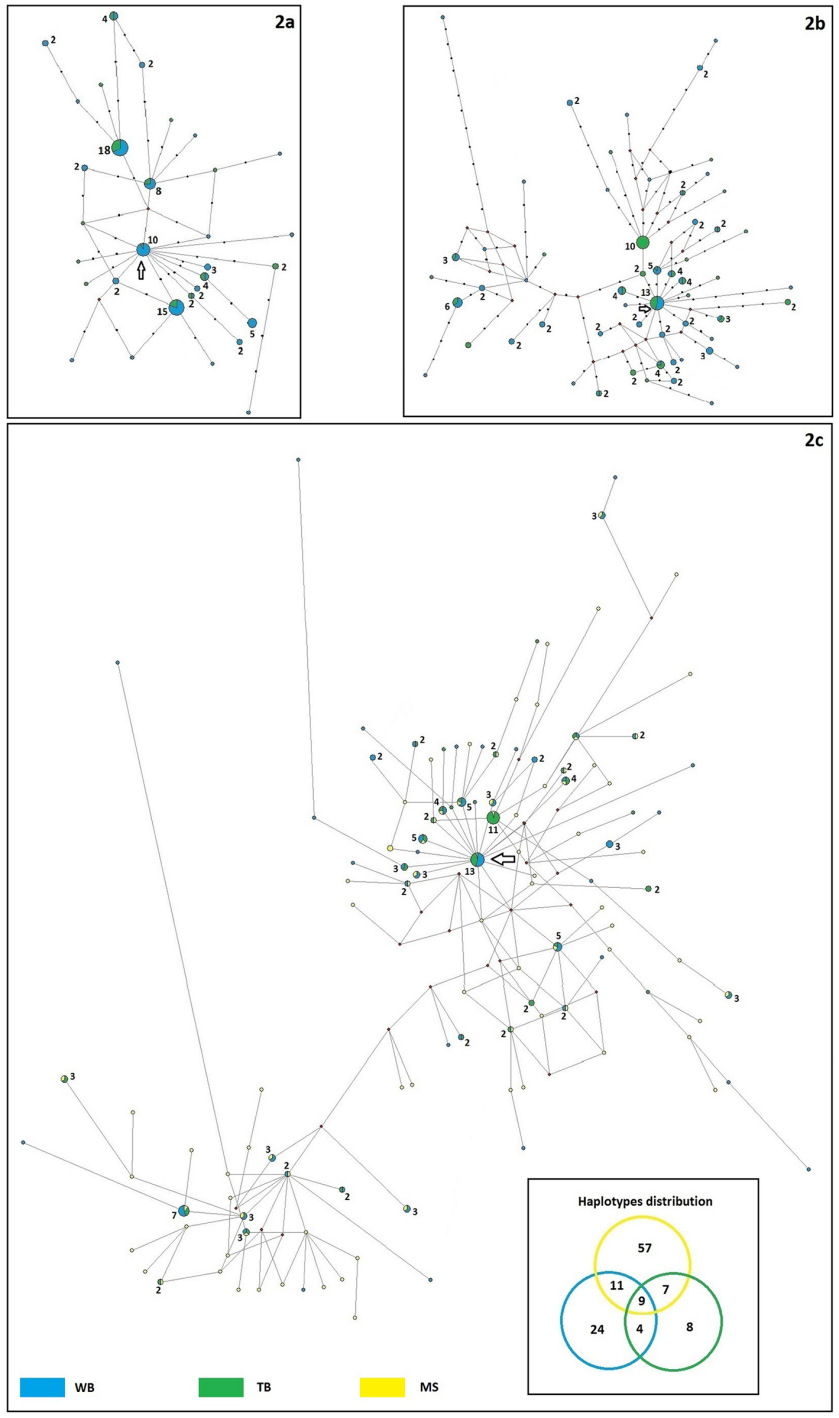
Haplotype networks. (A) *CYB*. (B) *DLOOP*. (C) *DLOOP* with MS haplotypes. Numbers indicate haplotype frequency (when not written, haplotype frequency = 1). Arrows indicate star shape. Black dots show mutational steps.

**Fig 3:**
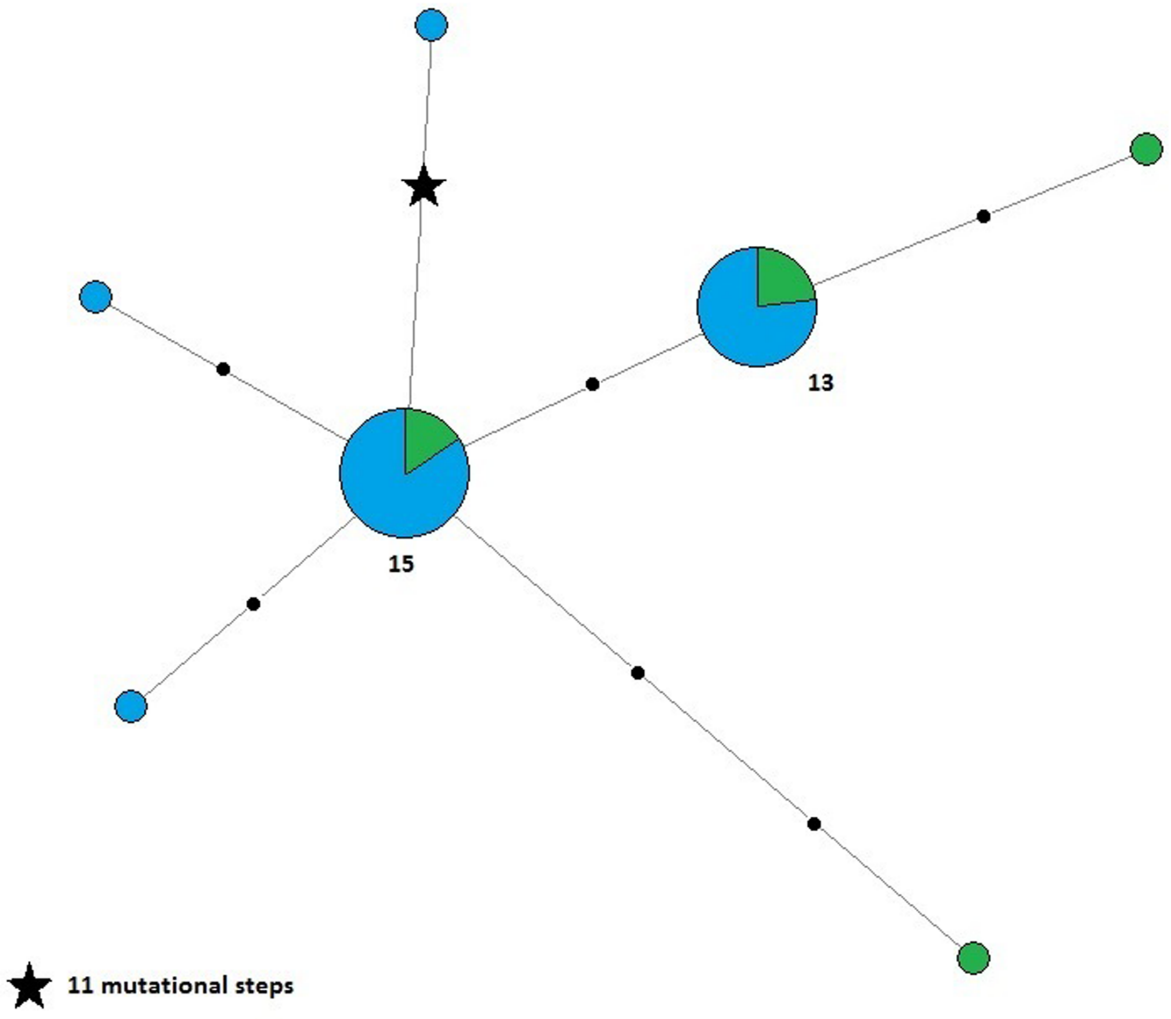
Haplotype networks for *SRY*. Numbers indicate haplotype frequency (when not written, haplotype frequency = 1). Black dots show mutational steps.

The mtDNA networks showed a consistent proximity between WB and TB colonies, for both fragments, as shown in Fig 2A and 2B. Seven *CYB* and 12 *DLOOP* haplotypes were shared between the two colonies, and the distances between nodes were usually in a range of one to four mutational steps. However, the majority of haplotypes were exclusive to either one of the colonies, as shared haplotypes always represented less than 25% of the total (22.6% for *CYB* and 21.1% for *DLOOP*, respectively). In the network with the 84 *DLOOP* sequences from MS (Fig 2C), 50% of all haplotypes were exclusive of WB, 28.5% of TB and 67.8% of MS, and only one haplotype was present in WB, with more than four mutational steps away from the closest haplotype (Fig 2C). All networks showed star-like patterns that spanned one or two mutations from the respective centre (small arrow). The network of *SRY* from Y chromosome was also star-shaped, as it consisted of a unique large star, containing all the haplotypes found in the samples. Only one male showed 11 mutational steps (Fig 3).

The percentage of missing microsatellite data was less than 4% over 17 loci, for both colonies. Sixty-eight per cent of individuals were entirely genotyped, and 28% not typed at only one or two loci. Missing data were equally distributed over loci.

**Fig 4:**
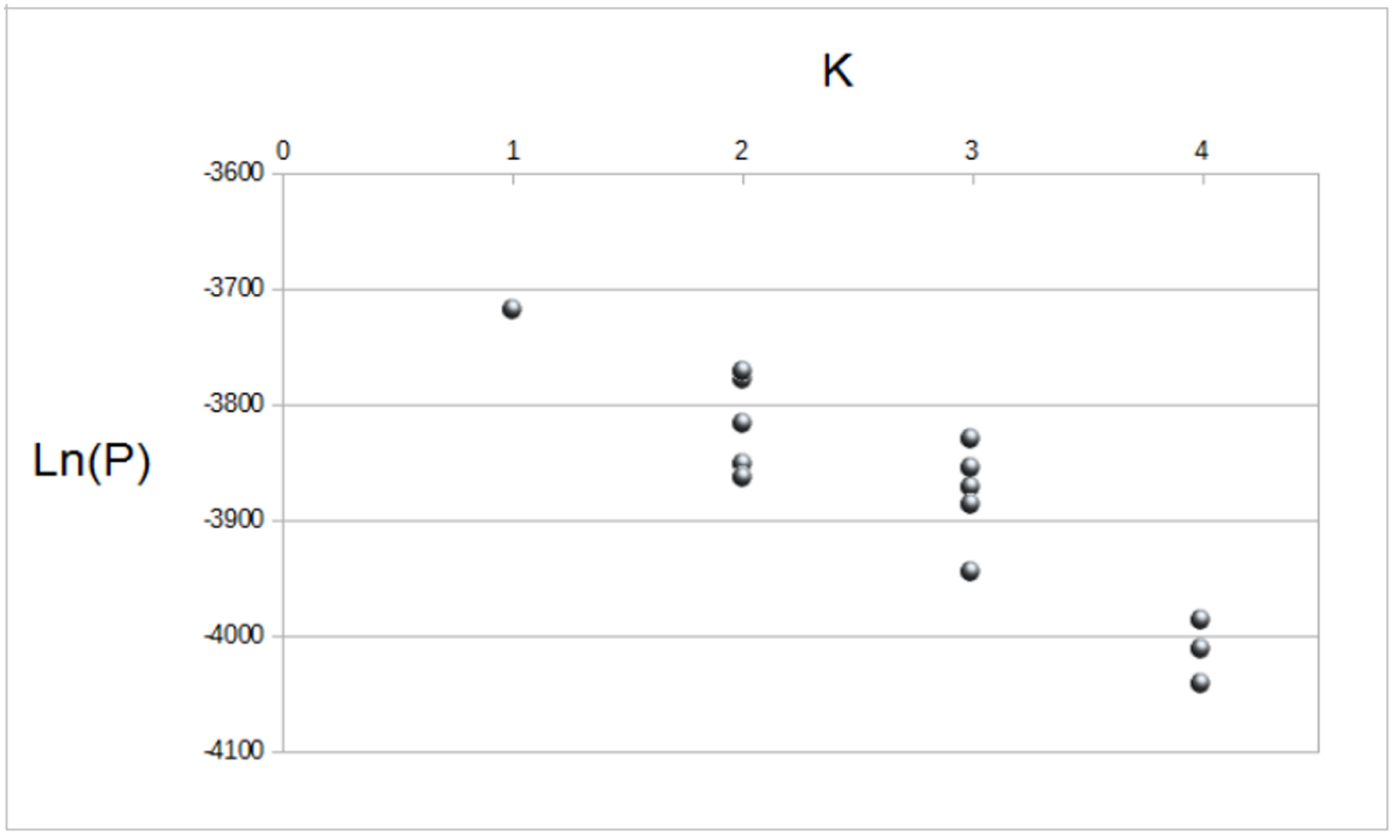
Likelihood values for each possible number of clusters (*K*) in Weddell seal colonies of WB and TB. Values are given in logarithm of the posterior probability (Ln(*P*)).

When pups and their known mothers were tested for maternity with Cervus, all pairs matched. Sixteen loci were polymorphic, ranging from two to 12 alleles per locus (mean = 7 ± 3.44). Locus HL2 was not polymorphic and HL4 deviated from Hardy-Weinberg equilibrium after Bonferroni’s correction (*p* < 0,001), due to an excess of homozygotes; therefore, these two loci were not used in the analysis. The other loci did not show excess of homozygotes or heterozygotes (thus, we could use the infinite sites model of DNA substitution mutation [65]) and also presented low proportion of null alleles (< 0.10). Expected and observed heterozygosities and F_is_ values were similar between colonies, suggesting that they have experienced the same low level of inbreeding. All parameters are shown in Table 4. Moreover, WB and TB colonies did not show a geographically partition of their genetic variation (*F*_*st*_ = 0.005, *p* > 0.05). The analysis with Structure found *K* = 1 as the most likely number of clusters, confirming that no structure was detectable between colonies, as shown in Fig 4.

**Table 4:** Microsatellite diversity indices.

	AR	*H*_*e*_	*H*_*o*_	*F*_*is*_
WB	6.53 ± 2.78	0.658 ± 0.206	0.645 ± 0.218	0.042 ± 0.096
TB	6.50 ± 2.65	0.629 ± 0.238	0.625 ± 0.252	0.049 ± 0.089
WB + TB	7.53 ± 3.24	0.650 ± 0.220	0.630 ± 0.220	0.041 ± 0.084

AR = allelic richness, *H*_*e*_ = expected heterozygosity, *H*_*o*_ = observed heterozygosity, *F*_*is*_ = inbreeding coefficient. Values are shown as mean ± stantard deviation

### Recent population expansion

**Fig 5:**
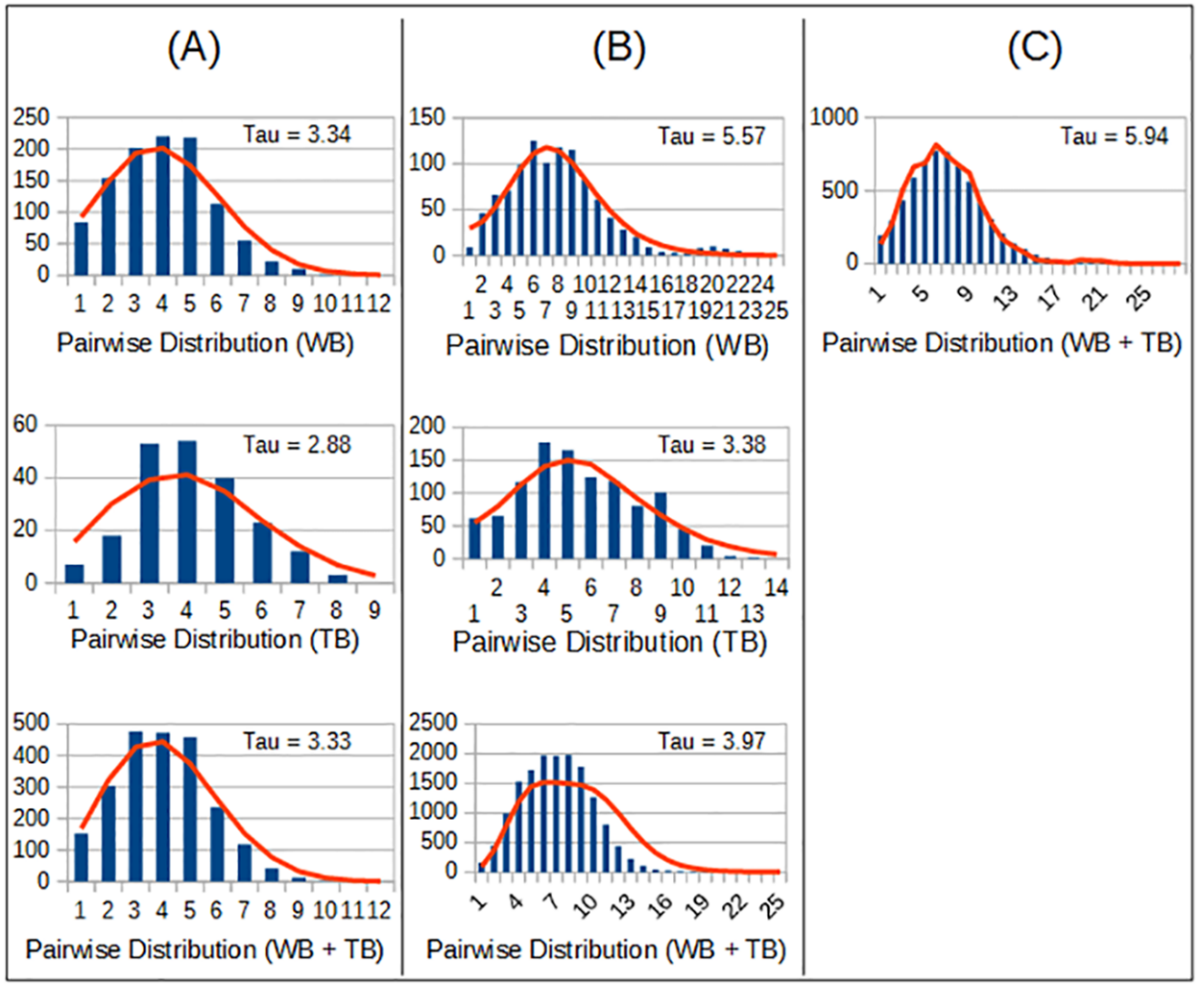
Distribution of pairwise nucleotide differences for Weddell seal colonies from Terra Nova Bay (TB) and Wood Bay (WB). (A) *CYB*. (B) *DLOOP*. (C) *SRY*. The bars show the observed distribution of pairwise differences, while the line represents the modelled distribution for sudden population growth. The peak of each distribution is shown as Tau (*Τ*). All graphics show a conformance to the sudden growth model.

The colonies showed a unimodal distribution of pairwise nucleotide differences for both fragments, indicating a sudden population growth (Fig 5), with *p* > 0.05. Similar pattern was also observed when the colonies were analysed together. The peak (*Τ*) of each distribution indicated similar dates of population expansion, using both *DLOOP* and *SRY*. Surprisingly, these values were twice as large as those obtained from *CYB*. The sudden expansion for WB + TB was also confirmed by Fu's *F*_*s*_ estimate, whose value was negative for all genes (*p* < 0.02), and reinforced by low raggedness values (Table 5).

**Table 5:** Analysis of recent expansion of Weddell seal colonies TB and WB.

Fragment	Index	WB	TB	WB + TB
*CYB*	Fu*F*_*s*_	-10.1	-8.8	-20.4
	*Τ*	3.34	2.88	3.33
	Years (generations)	124,813 (13,868)	107,623 (11,958)	124,438 (13,826)
	Mismatch SSD	0.002	0.005	0.002
	Raggedness	0.022	0.038	0.026
*DLOOP*	Fu*F*_*s*_	-19.9	-14.3	-42.2
	*Τ*	5.57	3.38	3.97
	Years (generations)	60,380 (6,708)	49,415 (5,490)	58,040 (6,448)
	Mismatch SSD	0.002	0.002	0.001
	Raggedness	0.006	0.011	0.006
*SRY*	Fu *F*_*s*_	//	//	-4.3
	*Τ*	//	//	5.94
	Years (generations)	//	//	48,545 (5,393)
	Mismatch SSD	//	//	0
	Raggedness	//	//	0.005

Fu*F*_*s*_ = Fu's test, *Τ* = peak of pairwise distribution, SSD = sum of square deviations.

### Effective population size

Estimations of *Θ* for both colonies, using the mitochondrial and nuclear gene fragments, are shown in Table 6. As results from LAMARC were very similar, only those from Migrate are shown (as mean values for ten runs). The estimates of the separate runs of *Θ* had standard deviation < 0.002.

**Table 6:** Values of *N*_*e*_ for Weddell seal colonies from TB and WB, based on *Θ*.

Fragment	Index	WB	TB	WB + TB
*CYB*	*Θ*	0.021 ± 0.001	0.020 ± 0.000	0.028 ± 0.001
	*N*_*ef*_	38,888	37,037	51,852
*DLOOP*	*Θ*	0.061 ± 0.001	0.043 ± 0.001	0.073 ± 0.002
	*N*_*ef*_	45,185	31,851	54,074
*SRY*	*Θ*	//	//	0.00461 ± 0.000
	*N*_*em*_	//	//	3,659

Based estimation of Θ for each colony were calculated with Migrate. The effective population numbers *N*_*ef*_ from *CYB* and *DLOOP* and *N*_*em*_ from *SRY* were estimated from *Θ*.

*N*_*e*_ estimates with microsatellites were larger with the program LDN_e_ than with N_e_Estimator (5% larger for WB, 101% for TB and 44% for WB + TB). When considering the two sampling sites as a single colony (WB + TB), *N*_*e*_ was much larger than the sum of the estimates obtained for each site (57% and 79% larger, with LDN_e_ and N_e_Estimator, respectively) (Table 7).

**Table 7:** Estimates of the effective population size for WB and TB, and for both colonies together.

*N*_*e*_	WB	TB	WB + TB
LDN_e_	706	372	1930
N_e_Estimator	668	185	1341

## Discussion

### Phylopatry and habitat choice

In the study of Curtis *et al*. [4] a large number of haplotypes was found in Weddell seals from MS, with a haplotype diversity of 0.98. This colony has been studied for the last few decades, but this paper represents the very first genetic study on Weddell seals that breed in areas of the Ross Sea other than MS. In our analyses, haplotype diversity (*Hd*) of TB and WB was 0.93 for *CYB* and 0.96 for *DLOOP*, very similar values both to MS and between the two genetic markers, even if they show different mutation rates, indicating consistency in our analysis. Moreover, even with a relatively small sample size, our estimates are close to those reported for other Antarctic seal species, such as *Lobodon carcinophaga* and *Ommatophoca rossii*, (*Hd* = 0.99 in both cases [4]). Furthermore, comparing these values with other pinnipeds with a wide range of distribution, they display a generally lower *DLOOP* variability, as is the case of *Otaria flavescens* in Brazil (*Hd* = 0.670 [66]) and the *Phoca vitulina* in the Arctic area (*Hd* = 0.363–0.943 [67]).

Surprisingly, 77% of *CYB* haplotypes of WB were exclusive of the area, and 44% were of TB. These proportions are even more impressive for the *DLOOP* fragment (81% and 58%, respectively for WB and TB), whose network showed exclusive haplotypes also when the sequences from MS were included in the analysis.

Weddell seals depend on cracks in the ice: they spend most of their life in the water, gaining access to land through already open cracks, or using their teeth to rasp the ice and open small holes to breathe [68]. Their migration among colonies is restricted, as individuals prefer to overwinter near their natal sites, and there might be physical/environmental limitations to their movment between breeding areas in winter. In this context, the isolation of some haplotypes in our analysis supports the philopatric behaviour of *L*. *weddellii* in the colony of MS, found by Cameron *et al*. [25]. In their work, they observed that the probability of a seal to return to breed to the same colony increased with age, to about age 12; at the same time, females with higher degree of site fidelity were more likely to have a higher reproductive rate. The strong breeding site fidelity in this species seems to be confirmed also in our study, as even at short distances (TB and WB are less than 100 km apart, while MS is around 300 km south) seals showed site preferences for reproduction. This level of site fidelity is nevertheless surprising, considering the potential capacity of dispersal of this species, where individuals can travel for more than 400 km (sometimes more than 1,500 km) during foraging trips [69], [70]. Moreover, fast ice is not a stable environment and can change across seasons, mainly because of strong wind currents in the region [20], and this precariousness can affect individual site fidelity, with seals changing reproductive site when the extent of ice coverage was larger the previous year [71]. Indeed, the three Antarctic pack ice species (Ross, crabeater and leopard seals) did not show evidence of population structure at microsatellite markers, suggesting a high rate of gene flow even among distant sites in all the three species [26], and crabeater seals usually use the floating pack ice for breeding and moving thousands of km [72].

Besides Weddell seals, some non-mammalian species inhabiting the Ross Sea also exhibit strong level of site fidelity. Adélie penguins (*Pygoscelis adeliae*) show a high degree of natal and breeding site fidelity during periods of stable conditions [73], [74], while Emperor penguins (*Aptenodytes forsteri*) can travel for more than 2,000 km to return to natal sites in the Ross Sea on pack ice to breed, after the moulting season [75]. This demonstrates that the tendency of some species for site fidelity can persist despite the dynamics of the Ross Sea landscape.

### Recent expansion of Weddell seals

The population expansion of Weddell seals in the Ross Sea had already been estimated in a previous study by Davis *et al*. [26]. However, data were obtained only for the colony of MS, and so our analyses on TB and WB can only improve the knowledge of Weddell seal expansion in the Ross Sea. The pairwise distribution of nucleotide differences for *CYB*, *DLOOP* and *SRY* was unimodal for both colonies, indicating a recent population expansion, that was supported by the star-shaped pattern in the haplotype networks. Fu's *F*_*s*_ test was significant only when considering WB and TB together (*p* < 0.02), which confirms that both colonies experienced an expansion as one large population. Unfortunately, we were not able to identify the originating point of this expansion, as the central haplotypes of the star-shaped networks were not exclusive of a specific colony, and haplotype networks cannot be used for time inference. However, time estimates for *CYB* suggested that the expansion might have started around 124,000 years ago. This estimation was much more recent when calculated with *DLOOP* or *SRY* markers (around 58,000 and 48,545 years ago, respectively). As these estimates refer to a generally recent expansion, we decided to use *DLOOP* value, since its mutation rate is higher and the results would be more precise in this context [76].

According to our results, the Weddell seal population of the Ross Sea has increased in abundance since the last recent glaciation. Starting one million years ago, temperatures oscillated between warmer and colder periods on an approximately 100,000 years cycle. The last movements of fast and pack ice in the area might have provided a larger and better habitat for breeding, promoting range and population expansion [77]. Comparing our new data with those from a *DLOOP* analysis by Davis *et al*. [26], the population expansion in our colonies seems to have occurred more recently than in MS (around 81,000 years ago). One possible explanation of this temporal discrepancy is the “south-to-north” direction of the pack ice rising during glacial periods. Hall *et al*. [78] studied mummified southern elephant seals (*Mirounga leonina*) from Late Holocene in the same area, and concluded that the decline of the species in the Antarctic during the last millennia followed that direction. As elephant seals inhabit today only ice-free beaches, the emerging of the pack ice might have been one of the reasons of their decline. If this pattern of population expansion also occurred during the Late Pleistocene, it might have influenced the distribution of Weddell seals, and their expansion might have started in MS (south) towards WB (north).

The Ross Sea is the most biological productive region in the Antarctic, and its shelf ecosystem is the most known and preserved stretch of the Antarctic seas, perhaps mainly due to its isolation from human interference [79]. The region comprises just 2% of the Southern Ocean, but it is responsible for 30% of its primary production [80], being thus very suitable for upper-trophic-level predators and robust food webs [81]. The entire region is a key area for many species that inhabit Antarctica. Due to its richness in biomass and seal preys, it seems reasonable to assume that the Weddell seal population of the Ross Sea has not yet reached its maximum.

### Effective population size: Differences between nuclear and mitochondrial markers

Analysing Weddell seals from MS, Davis *et al*. [26] found significant genetic differentiation only between sites more than 700 km apart. Similarly, studies on underwater vocalizations found differences in seal vocal repertoire only between colonies at the same minimal distance [82–84]. The present work analysed different colonies from the Ross Sea, but even in this case microsatellite analyses suggested low genetic structure between WB and TB colonies (low *F*_*is*_ and *F*_*st*_, *K* = 1, see results for details). These results were somehow expected, given the proximity between the colonies (around 90 km), which makes them part of a single population.

Microsatellite *N*_*e*_ was estimated as 1,930 and 1,341 individuals, with LDN_e_ and N_e_Estimator, respectively. The difference between estimates is not unsurprising, as the two programs have two different ways to deal with missing data [59], [60]. In the end, wepreferred to use the value from N_e_Estimator, due to its more complex and conservative calculation method [60].

For the entire Ross Sea population, the *N*_*ef*_ estimates using mitochondrial fragments and different software were very similar, indicating 48,000–54,000 females that genetically contribute to the next generation. This range is very close to a previous estimate made by Davis *et al*. [26] in the MS colony (around 55,000 females). Regarding males, our *N*_*em*_ estimates using *SRY* gene was 3,143, and would represent 5.97% of *N*_*ef*_. This is the first male estimate for Weddell seals, and would mean a male/female ratio between 1:12 and 1:16, a very unbalanced value, given the life-history traits and mating system of the species. Most likely, this discrepancy is due to our small male sample size. In fact, several phocids show a breeding sex ratio (i.e., number of females per breeding male) biased towards females, as a consequence of their mating system. Nevertheless, the level of polygyny differs among species, from those with a slightly polygynous mating system (e.g., *Pusa hispida* (1:2.4) [85]) to *M*. *leonina*, which is among the most polygynous species of all mammals and shows a breeding sex ratio of 1:14.5 at the Falklands’ colony [86]. In general, Weddell seals have a medium level of polygyny; a paternity study on Big Razorback Island (Erebus May, MMS) found an average of 2.54–3.33 pups sired per successful male over two breeding seasons [54]. Although the level of polygyny in Weddell seals is higher than those in other acquatically mating phocids, e.g. hooded seal (*Cystophora cristata*) [87] and crabeter seals (*L*. *carcinophaga*) [88], a breeding sex ratio of 1:16 is highly unlikely, and more efficient strategies should be adopted to sample males and achieve a more realistic estimate. Among mammals, the proportion between *N*_*e*,_ based on both males and females, and census size is around 0.45 [89]. This low proportion is due to several life-history and demographic traits, such as fluctuating population size, breeding sex ratio, overlapping generations and spatial dispersion. In fact, for pinnipeds, the unbalanced male/female sex ratio is one of the most important factors that can reduce *N*_*e*_. In the present study, we could obtain total *N*_*e*_ according to two hypotheses:

1. Weddell seal adult sex ratio is 1:1. In this case, our *N*_*e*_ would be 2 x *N*_*ef*_ (around 213,000–240,000), and census size would be around 192,000–216,000 individuals (as the result of *N*_*e*_ multiplied by 0.45 [89]). This seems a reasonable estimate, because it corresponds to 30% of the Weddell seal census size of Antarctica (730,000–800,000 [3], [5]), and the Ross Sea coast covers 20% of the Antarctic Sea shore;
2. Weddell seal adult sex ratio is unbalanced. In this second scenario, we would use the *N*_*em*_ of this study, and apply the formula *N*_*e*_ = 4 x *N*_*ef*_ x N_em_ / (*N*_*ef*_ + *N*_*em*_) for unbalanced sex ratios [90]. Following this hypothesis, *N*_*e*_ for the Weddell seal population of the Ross Sea would be 13,708–13,922, and census size would be much smaller (30,462–30,938 individuals) than in the first hypothesis. This second estimate seems largely unlikely, and highligths the need of an appropriate adult sex ratio estimate.

## Conclusions

The Weddell seal is a very important mammal from the Antarctic continent and plays a key role in the Southern Ocean ecosystem. Due to the absence of predators (including humans), its populations have experienced recent expansions since the last glacial cycle. The number of reproductive females we estimated as reproducing in the Ross Sea is higher than the previous census size for the area, showing that the actual number of individuals might have been previously underestimated. Mitochondrial analysis showed a consistent genetic variation between the colonies, even if they are close to each other, supporting the evidence of site fidelity in the species, even over short distances. Nevertheless, both analysed colonies, along with those from McMurdo Sound, seem to be part of a larger population inhabiting the Ross Sea.
